# Chronic conditions in children and young people: learning from administrative data

**DOI:** 10.1136/archdischild-2016-310716

**Published:** 2016-05-31

**Authors:** Linda P M M Wijlaars, Ruth Gilbert, Pia Hardelid

**Affiliations:** 1 Children's Policy Research Unit, UCL Institute of Child Health, London, UK; 2 Farr Institute of Health Informatics Research London, London, UK; 3 Department of Primary Care and Population Health, University College London, London, UK

**Keywords:** Data Collection, Epidemiology, General Paediatrics, Health services research

## Introduction

Over the last century, the primary burden of disease in children and young people has shifted from infectious diseases towards chronic conditions.^1^ Improvements in neonatal and paediatric care for chronic conditions mean more children with previously lethal conditions are now surviving into adulthood.^2^ ^3^ Depending on the definition used, 13–27% of children are affected by chronic conditions.^4^ Chronic conditions affect many aspects of the lives of children with consequences that endure into adulthood.^5^

Quality of healthcare for children with chronic conditions is a research and policy priority, but comes at a cost. In the USA, it is estimated that children with serious, complex chronic conditions account for 10% of admissions, but 41% of hospital charges.^6^ As life expectancy increases, these costs extend into adulthood. Better quality of healthcare during childhood can improve educational achievement and employment prospects and reduce disability and dependency in adulthood. To find out whether improvements in healthcare are indeed improving long-term outcomes for children with chronic conditions and their families, we need data.

In this article, we review how administrative health data can be used to evaluate the frequency of healthcare utilisation by children affected by chronic conditions and to evaluate outcomes. Administrative health data are collected routinely for non-research purposes, including for patient or service management or financial reimbursement. We focus specifically on hospital administrative databases and vital registration systems. The evidence generated by administrative hospital data on the burden and type of chronic conditions can help with planning and design of services and can be used to determine (the effects of) changes in practice of policy. We also discuss how studies using administrative health databases could be extended through data linkage, to other sectors, such as education and social care, to measure outcomes in adulthood, and to data for the parents or carers, to evaluate impacts on wider aspects of children's lives.

## Evaluating chronic conditions in hospital administrative data

Evidence on healthcare utilisation is rapidly emerging as a result of the improving quality, standardisation and longevity of administrative healthcare data. In Western Australia, Canada, Scotland and the Nordic countries, administrative healthcare data have been routinely collated over decades, making it possible to evaluate patterns of healthcare for children with chronic conditions from birth to adulthood at a fraction of the cost of traditional cohort studies.^7–11^ Of all types of healthcare data, hospital administrative data, which primarily record hospital activity for reimbursement and commissioning purposes, are the most standardised and comprehensive across many healthcare systems and they are usually longitudinal, meaning that episodes of care for the same patient are linked over time. Many of these databases record diagnoses in the form of International Classification of Diseases (ICD) codes. In the National Health Service (NHS), these codes are entered by professional coders based on hospital discharge records.^12^ We can use these codes to identify chronic conditions in hospital administrative data to measure the changing burden of chronic conditions over time, between countries and between regions or hospitals.^13–15^

**Figure 1:**
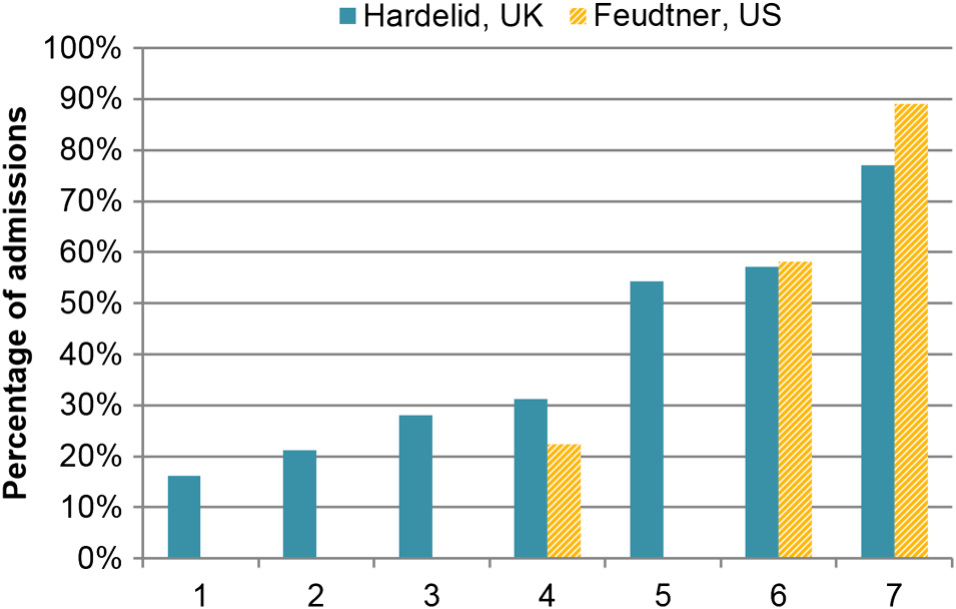
Proportion of children admitted to hospital with chronic conditions (papers used for the UK^21^ ^24–26^ and USA^7^ ^23^ ^27^) groups: (1) children with emergency admissions for accident-related injuries; (2) children with emergency admissions for adversity-related injuries (drug/alcohol use, self-harm or violence); (3) all hospitalised children; (4) children with a first emergency admission; (5) children with a laboratory-confirmed bloodstream infection; (6) children who died; (7) children with four or more emergency admissions in 1 year. All UK papers used the ‘look-back method’ and included hospitalisations records from the 12 months prior to the index admission to define whether children had a chronic condition. US papers only included diagnosis codes entered for the index admission.

Different classifications for chronic conditions in children have been developed in the UK and the USA (box 1), though both produce similar proportions of chronic conditions for some groups of children (figure 1). These classifications offer a starting point for studying chronic conditions in children, but more studies are needed that validate these and other classifications and how they vary.

Box 1 **Classifications to identify chronic conditions in hospital administrative data**

**Hardelid classification**^19^

*Definition of chronic condition*: Any health problem requiring clinical follow-up for >12 months in 50% or more of cases. Medical follow-up was defined as repeated hospital admission, specialist follow-up through outpatient department visits or use of support services such as physiotherapy or speech and language therapy.

*Data sources*: Hospital administrative data for England and death registrations.

*Coding system*: International Classification of Diseases (ICD)-10 codes, grouped into eight mutually exclusive body system categories and including additional generic codes (eg, gastrostomy feeding). Examples of conditions include cerebral palsy, epilepsy, learning disability, asthma, self-harm.

*Development*: Sources of ICD codes for chronic conditions were (1) a literature review of studies using ICD coding for chronic conditions in children using administrative health databases; (2) a systematic search of all ICD-10 codes; (3) analysis of codes in longitudinal cohorts of children with known chronic conditions to detect codes not previously included; and (4) examination of codes recorded for children with no apparent chronic conditions. The list of codes and groupings were reviewed by an independent panel of clinicians, and a further panel of experts in the epidemiology of chronic conditions in children using administrative health databases.

**Feudtner classification**^20^

*Definition*: Any medical condition that can reasonably expected to last at least 12 months (unless death intervenes) and to involve either several different organ systems or one organ system severely enough to require specialty paediatric care and probably some period of hospitalisation in a tertiary care centre.^21^

*Data sources*: Originally developed for use on cause of death on death records, but has subsequently been applied in hospital admission and emergency department data.

*Coding system*: First classification was developed using ICD-9 codes, but has now been updated for ICD-10. The codes are grouped into 10 (previously 9) groups based on body system or disease origin (eg, neonatal conditions). Examples of conditions include cerebral palsy, epilepsy (not including benign childhood epilepsy (G40.0) and petit mal (G40.7)), organ transplantation, dependence on devices. Does not include asthma or mental health problems such as depression or self-harm.

*Development*: The original classification was developed using the following steps:^22^ (1) development of initial list (based on clinical experience); (2) literature review of studies identifying children with high-cost conditions; and (3) initial analyses of cause of death in children without complex chronic conditions.

A strength of longitudinal hospital administrative data is that children with chronic conditions can be identified in repeated attendances even though their condition might not be recorded at every admission. One limitation is that chronic conditions that rarely require hospital admission will not be captured in hospital administrative data. A second limitation is that the accuracy of hospital data depends on data quality checks, which depend on the primary purpose of the data. For instance, in England, Hospital Episode Statistics data are collected to determine hospital reimbursement. Coding of diagnoses and procedures that determine costs are well recorded (84% and 97% for procedures and primary diagnosis compared with case notes, respectively).^16^ Variables not linked to reimbursement, such as ethnicity (79% complete),^17^ are less likely to be complete or accurate.

In the next section, we provide examples of how, despite these limitations, validated algorithms can be used to characterise chronic conditions in administrative data with sufficient accuracy for population-based analyses for service evaluation or research.^18^

### Chronic conditions in children who die

**Figure 2:**
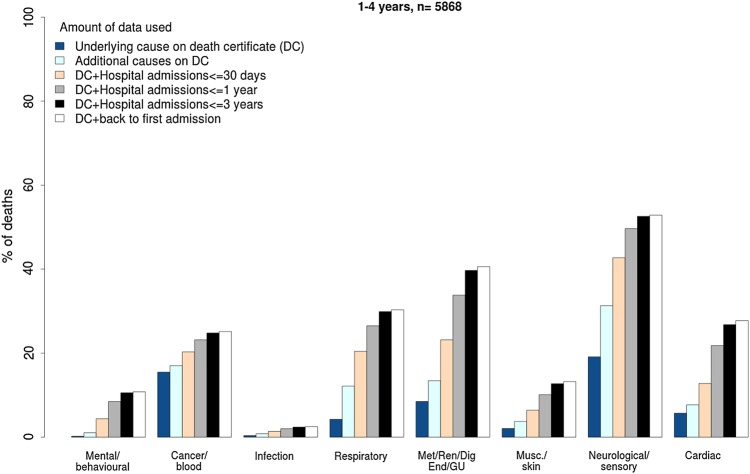
Proportion of children who died aged 1–4 years with a chronic condition in England and Scotland (2001–2010) and Wales (2003–2010), according to the type of chronic condition and amount of linked data used. GU, genitourinary.

We used hospital administrative data linked to death certification data for children who died aged 1–18 years in England, Scotland and Wales to investigate the proportion of one or more chronic conditions using the Hardelid classification (see box 1 and online supplementary appendix B).^20^ When analyses were based on the cause of death on the death certificate, the proportion of children who died with chronic conditions was just 57%. Looking back at all admissions over the previous year, the proportion with a chronic condition increased to 71%.^21^ This ‘look back’ strategy had a small effect on the proportion of children who died with cancer, but made a big difference to the proportion who died with neurodevelopmental conditions (figure 2). This approach helps to focus preventive strategies on patient groups who contribute the largest number of child deaths.

10.1136/archdischild-2016-310716.supp1 Supplementary data

Similar studies have been conducted in the USA using the Feudtner classification (box 1),^22^ although without the ‘look back’ assessment of hospital admissions in the year before death.^23^ Using cause of death, Feudtner reported complex chronic conditions in 58% of child deaths between 1 and 18 years old in 1997, similar to the UK results based on death certification data alone (figure 1).

### Chronic conditions in other patient groups

We used the Hardelid classification to measure the proportion of chronic conditions in several other patient groups admitted to the NHS in England (see figure 1 and online supplementary appendix A). Based on the index admission and any admissions in the previous year, the proportion of codes indicating a chronic condition increased with age and with the clinical severity of the patient group (see figure 1, table 1 and online supplementary appendix A).

**Table 1:** Proportion of children affected by a chronic condition in various hospital settings

Study	A*			B		C	D	
	Children who died in 2006–2010	Children who died in 2006–2010 (excluding injury deaths)	All children hospitalised in 2006–2010	Children's first emergency admission in 2011	Children with ≥4 emergency admissions between 2010 and 2011	Children with a bloodstream infection 2007–2011	Children with emergency admission for adversity-related injury in 1998–2011	Children with emergency admission for accident-related injury (no adversity) in 1998–2011
Age group	% n/N	% n/N	% n/N	% n/N	% n/N	% n/N	% n/N	% n/N
<1 year	–	–	–	–	–	**51.6**† 2283/4422	–	–
1–4 years	**67.1** 1688/2516	**75.7** 1605/2121	**24.4** 208 186/853 557	**18.0** 28 228/156 646	**67.9** 4157/6124	**55.4** 3080/5565	–	–
5–9 years	**72.9** 1028/1410	**82.6** 993/1202	**27.9** 179 862/645 047	**28.1** 23 253/82 894	**89.1** 1728/1940	**64.0**‡ 396/619	–	–
10–14 years	**64.6** 1078/1669	**80.3** 996/1241	**28.1** 158 425/563 504	**35.5** 28 247/79 618	**85.3** 1732/2031	–	**21.2** 70 874/333 733	**16.1** 104 903/651 494
15–19 years	41.3* 1522/3686	**72.9** 1288/1767	32.0* 257 877/805 046	**48.0** 56 452/117 659	**81.1** 3140/3872	–		
Total	**57.2** 5316/9281	**77.1** 4882/6331	**28.1** 804 350/2 867 154	**31.2** 136 180/436 817	**77.0** 10 757/13 967	**54.3** 5759/10 606	**21.2** 70 874/333 733	**16.1** 104 903/651 494
Change youngest—oldest group	*−25.8*	*−2.8*	*7.6*	*30.0*	*13.2*	*12.4*	*n/a*	*n/a*

The boldface is used to distinguish relative (percentage) measures from absolute measures. And italics are used to emphasise the total row.

*Results from study A only up to age 18 years inclusive.

†Results from study C only for children discharged home from birth and age 1 month to 1 year on admission date.

‡Results from study C only for children aged 5 years rather than 5–9 years. n/a, not applicable.

#### Injury

We found a relatively low proportion of chronic conditions recorded for adolescents (aged 10–19 years) admitted for injury. Chronic conditions were recorded in the past year for 16% of accident-related injury admissions to 21% of adolescents admitted for adversity-related injury (figure 2 and table 1). Adolescents with chronic conditions had a threefold higher risk of death or repeated emergency admission over the subsequent 10 years.^23^ Chronic conditions affected 55% of children with laboratory-confirmed bacteraemia in a study using linkage between national infection surveillance and admission data.^24^

#### Frequent emergency admissions

Our analyses of all emergency admissions of children to the NHS in England revealed that 77% of children who had four or more emergency admissions had chronic conditions based on any codes recorded during hospitalisations within the last 12 months (figure 1).^25^ This predominance of chronic conditions in children with repeated emergency admissions was also found in a US study using the Feudtner coding list.^26^

#### Ambulatory care-sensitive conditions

Among US children admitted with primary diagnoses that met the criteria for ‘ambulatory care-sensitive conditions (ACSCs)’, 40% had chronic conditions recorded within the past year.^27^ ACSCs are defined as conditions where effective community care and case management is thought to help prevent hospital admission.^28^ The high proportion of chronic conditions suggests that many admissions categorised as ACSC may not be appropriately managed in the community because they are medically complex.

#### Readmissions

Studies in both the USA and UK have investigated chronic conditions in children who are readmitted as an emergency. In both countries, hospitals are penalised financially for emergency readmissions that occur within 30 days of a previous discharge based on the rationale that emergency readmission is a sign of poor quality care at the index admission. However, research in both countries shows that many children who have an emergency readmission within 30 days (19–71%) have records in the past year indicating one or more chronic conditions.^17^ ^18^ These findings suggest that financial penalties are reducing remuneration for readmissions for medical complexity, rather than poor quality hospital care, and may disincentivise appropriate and necessary care for children with chronic conditions.

Our analyses of readmissions in the NHS in England found that children with chronic conditions were often admitted with different primary diagnoses that related to a different organ system or symptoms rather than their chronic condition.^29^ Similar results have been reported for adults.^30^ These findings suggest that children with chronic conditions need a holistic view of their healthcare needs and may not always follow condition-specific care pathways.

## Future research directions

We have outlined examples of cost-efficient and policy-relevant research that can be undertaken using hospital administrative data. Much more can be done. First, more studies are needed that make use of the longitudinal record to follow patients' trajectories of healthcare, before and after diagnosis or interventions for chronic conditions to monitor quality of care and effectiveness of innovations. For example, research in England showed variation in outcomes of cardiac surgery in children as part of the Bristol enquiry into cardiac postoperative deaths.^31^ Hospitals can help by systematically recording information (including dates) on the implementation of new treatments, staffing configurations or changes in practice.

Second, interventions or changes in one sector of healthcare for children with chronic conditions are likely to impact on other parts of the service. To understand these spillover effects on the service, we need more widespread linkage of data between healthcare sectors. Linked primary care and hospital data are available for research in the form of the Clinical Practice Research Datalink. However, these data currently apply to only 4% of general practice registered patients in England, and the period of linkage is restricted to the period of registration, making it hard to measure long-term outcomes for children who move practices. In addition, access to the data is very expensive and beyond the reach of many researchers.^32^

Linking data between hospital and other health services for children with chronic conditions is also important, particularly for children using disability services or those using child and adolescent mental health services (CAMHS). In January 2016, the Health and Social Care Information Centre (HSCIC) started data collection for a national CAMHS data set, which is expected to be available in 2017 (http://www.hscic.gov.uk/camhs). These data will be linkable to hospital administrative data; however, it will take years for longitudinal data to accrue to allow follow-up of children's outcomes.

Third, we need linkage to parental information in order to evaluate child outcomes. Well-characterised, whole-country birth cohorts that combine maternal characteristics with longitudinal follow-up of child outcomes have been used for years in Scotland, Western Australia, Manitoba, Sweden and Denmark. Such linked data could be generated for England from the late 1990s onwards using probabilistic linkage methods to link hospital administrative data for birth episodes and by using clinical characteristics common to both mother and baby records. Such linkage would allow investigation of both mother and child trajectories of hospital care and could provide evidence about the impact of maternal chronic conditions, or pregnancy complications, on childhood outcomes. HSCIC could harness more evidence for children and families from the data it already holds by implementing probabilistic linkage methods.

### Beyond healthcare: linkage to other sectors

Beyond health, schools provide important social and educational support for children with chronic conditions. Linked longitudinal administrative data within education could be used to generate evidence on whether such support from schools improves educational attainment or reduces school exclusion. Linkage to employment data could enable assessment of job prospects for children with chronic conditions.^33^ Linkage to health data would enable evaluation of whether support by schools reduces use of health services. Extending linkage to include health, education and children's social care would be crucial to effectively plan and monitor services and to determine whether changes in practice improve education, social welfare and health outcomes for children with chronic conditions.

Linkage of administrative data across services for children and families for these services is established practice in the Nordic countries^9^ and has partly been achieved in Scotland.^34^ The Administrative Data Research Centre for England has been working over the past year to link education data and hospital administrative data for England as part of a study to evaluate school achievement and patterns of hospitalisation in children with chronic conditions. Key challenges are establishing permissions for linkage of administrative data across government sectors, the need for accurate linkage methods that do not rely on a universal personal identifier and the lack of R&D capacity within government data providers to work with the academic community to bring innovation to data processing.

Administrative healthcare data offer a major resource for national and international research into chronic conditions affecting children. Chronic conditions have long-lasting effects on children, the adults they become, their family and the next generation. Administrative data can also directly inform clinical practice by identifying variations in past healthcare histories and child outcomes, the effectiveness of interventions or changes in practices, or by linkage to clinical trials to enable long-term follow-up.^35^ The quality and longevity of UK healthcare data and the size of the population of children and their parents captured by these data have the potential to generate cost-effective and valuable evidence for families, for services and to advance scientific understanding of the development and consequences of chronic conditions. With adequate investment in developing innovative approaches to data quality and linkage, the UK could hold the world's largest and best-characterised child cohorts, based on administrative data. To achieve this, the HSCIC and other government data providers need to become part of the research and development cycle, devising new ways of using administrative data to improve service evaluation and research for public benefit.
