# Supplementary material for: Chronic conditions in children and young people: learning from administrative data
Source: Arch Dis Child. 2016 May 31;101(10):881–5. doi: 10.1136/archdischild-2016-310716 (PMC5050282; doi:10.1136/archdischild-2016-310716)
Supplement: Supplementary data [file archdischild-2016-310716supp.pdf]

Appendix A: Results from individual studies using the Hardelid chronic condition coding classification in Hospital Episode Statistics

We used data from four projects, which respectively focussed on children who died (Study A), had recurrent emergency admissions (Study B), had a bloodstream infection (Study C), or had an emergency admission for injury during adolescence (Study D). For Study C, we present 3 years of additional data not included in the original publication. Study A also included a control group of all children admitted to hospital in the English National Health Service (NHS).

Data source

The Hospital Episode Statistics (HES) database was set up for commissioning and costing services, covering all inpatient admissions in English hospitals paid for by the NHS. Individual patient records can be linked over time for admissions ending on the 1st April 1997 onwards to create longitudinal hospital trajectories for each child. The structure of HES,[8] and data cleaning algorithms used across the four projects have been described elsewhere.[9] Diagnoses in HES are recorded using International Classification of Diseases version 10 (ICD10) codes (http://apps.who.int/classifications/icd10/).

All HES data extracts were provided by the Health and Social Care Information Centre (HSCIC). Data in three projects were anonymised and therefore ethics committee approval was not sought;[4,5,7] Study C used patient identifiers to probabilistically link HES to Public Health England routine microbiology data[6] for public health surveillance purposes.

Defining chronic conditions

We identified children with CCs using a list of ICD10 codes developed specifically for identifying CCs in children and for use in longitudinal hospital data, which was verified by a panel of clinicians (Appendix B).[9] This list defines a CC as any health problem likely to require follow-up for more than one year, where follow-up could be repeated hospital admission, specialist follow-up, or use of support services such as physiotherapy.[2] All four studies defined children as having a CC if they had a CC code entered in any admission in the year prior to index admission.

Study population

Hospitalised paediatric populations are summarised in Table 1 and eFigure 1 and ranged from infants to 19 year olds. The start and end dates of the study period varied according to the study (eFigure 1).Study A and C include both emergency and elective admissions, whereas study B and D include only emergency admissions.

Study A – Chronic conditions (CCs) in children who died

This study characterised CCs in children who died and included CCs included on the death registration and hospital records. The study population for this study were children who died between 2001 and 2010, selected into the study through mortality records in England, Scotland and Wales. Mortality records were linked to a child's preceding hospital record to determine the proportion of chronic conditions using longitudinal hospital trajectories. Because of delayed registration of death, we included all deaths that occurred before the end of 2010 that were registered up to August 2012.

In the results presented here, any chronic conditions recorded on the death certificate were excluded to make the CC proportion estimate similar to the other studies. To provide a benchmark of the frequency of CCs in hospitalised children we also determined the proportion of CCs among all children admitted to hospital under the National Health Service (NHS) in England between 2006 and 2010 aged 1–18 years old. Using their most recent admission as the index, we determined the

proportion of children with one or more CCs recorded in their current or previous admissions during the preceding 12 months.

Study B – CC's in children with recurrent emergency admissions

This study included all children (0-18 years) who had an emergency admission in the 2009 financial year. When a patient had multiple admissions in 2009, we considered their first emergency admission as their index admission and counted all subsequent emergency admissions during two years of follow-up. We assessed whether children in the cohort were affected by CCs by scanning all diagnostic codes entered during each admission, comparing them to Hardelid et al.'s codes.

Study C – CC's in children with bloodstream infection

Laboratory reports of positive blood culture specimens in children (1 month – 5 years) voluntarily submitted to Public Health England's national microbiology database between 1 April 2007 and 31 March 2011 were probabilistically linked to all in-patient Hospital Episode Statistics (HES) paediatric cases from the same time period. Children less than one month old were excluded to minimise the numbers of vertically transmitted infections captured, and all children who had never been discharged from hospital were excluded. Only the index Blood Stream Infection (BSI) case occurring within the 4-year period was analysed. CCs recorded in the current and previous hospital admissions within one year were grouped using Hardelid et al.'s groupings.

Study D – CC's in adolescents with emergency admissions for injury

This project originally looked at the risk of death and emergency re-admission in adolescents hospitalised with emergency admissions for injuries related to either adversity (victimisation, self-harm or drug or alcohol misuse) or accidents. We studied 1 076 914 individuals who 1) were 10-19 years old in 1998-2011, 2) had at least one emergency admission for injury at age 10-19 (one was randomly selected as the index admission, for the 8% who had more than one), 3) could be followed up for at least one day after discharge and 4) were admitted for either adversity- or accident-related injury. For the current report, we present proportions of CCs in 1 080 181 individuals who meet criteria 1) and 2) but not necessarily 3) or 4). We present the proportion of individuals with a CC record at any admission at the index admission or in the preceding year for three groups: those who had at their index admission 1) adversity-related injury, 2) no adversity-related injury but did have accident-related injury, and 3) other causes of injury (no adversity or accident-related injury).

References

1 Health and Social Care Information Centre. HES Data Dictionary: Inpatients. 2010.http://www.hscic.gov.uk/media/1358/HES-Hospital-Episode-Statistics-HES-Admitted-Patient-Data-Dictionary/pdf/HES_Inpatients_DD_Sept10.pdf

2 Hardelid P, Dattani N, Davey J, *et al.* Overview of child deaths in the four UK countries. RCPCH 2013. bit.ly/hardelidreport

3 Hardelid P, Dattani N, Gilbert R, *et al.* Estimating the prevalence of chronic conditions in children who die in England, Scotland and Wales: a data linkage cohort study. *BMJ Open* 2014;**4**:e005331. doi:10.1136/bmjopen-2014-005331

4 Wijlaars L, Hardelid P, Woodman J, *et al.* Contribution of recurrent admissions in children and young people to emergency hospital admissions: retrospective cohort analysis of hospital episode statistics. *Arch Dis Child* in press.

5 Herbert A, Gilbert R, Gonzalez-Izquierdo A, *et al.* Ten-year risk of death and emergency re-admission in adolescents hospitalised with violent, drug/alcohol related, or self-inflicted injury: a population-based analysis. *PLoS Med* 2016.

6 Henderson KL, Müller-Pebody B, Wade A, *et al.* Timing of positive blood samples does not differentiate pathogens causing healthcare-associated from community-acquired bloodstream

infections in children in England: a linked retrospective cohort study. *Epidemiol Infect* 2014;:1–6. doi:10.1017/S0950268814003306

7 Simon TD, Berry J, Feudtner C, *et al.* Children with complex chronic conditions in inpatient hospital settings in the United States. *Pediatrics* 2010;**126**:647–55. doi:10.1542/peds.2009-3266

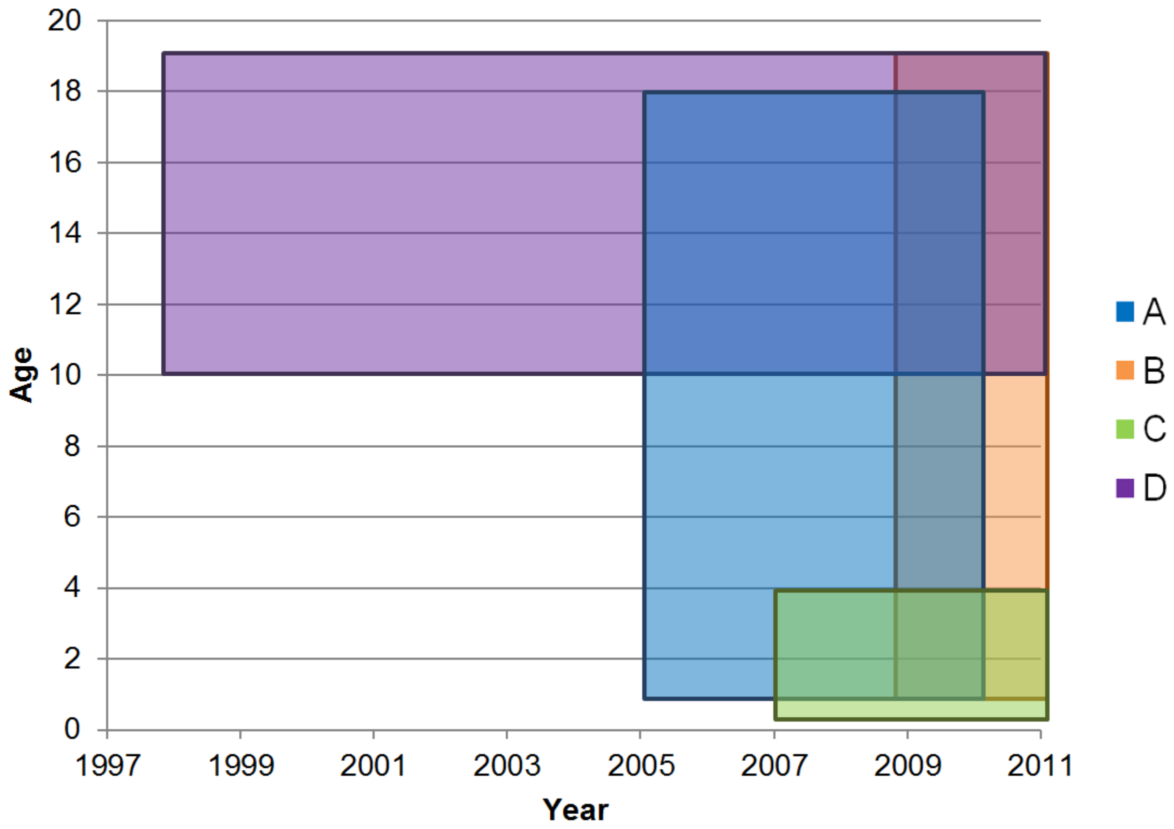

eFigure 1: Overlap in ages and years covered by the four included projects

eTable 1: Overview of hospitalised paediatric populations

**Project**	**Age group**	**Admitted during**	**Group**	**Follow-up**
A	1 –18 years	1 January 2005 - 31 December 2010	a) Children who died between 2006 and 2010<br><br>b) Children admitted to hospital at least once between 2006 and 2010[15]	1 year back from the date of death for a) and 1 year back from last admission in study period for b)
B	0 – 18 years	1 April 2009 – 31 March 2012	Children who had multiple emergency admissions over a 1-year period [20]	1 year back from last emergency admission in 2011-12 financial year
C	1m – 5 years	1 April 2007 - 31 March 2011	Children with a laboratory confirmed bloodstream infection[19]	1 year back from the date of the positive blood specimen

D	10 – 19 years	1 Jan 1998 – 31 Dec 2011	Adolescents with ≥1 emergency admission for injury between age 10 and 19, inclusive[18]	1 year back from index emergency admission for injury

Appendix B: International Classification of Diseases version 10 codes used to identify children with chronic conditions in English Hospital Episodes Statistics (HES) data

Type of chronic condition	Categories	Codes
Mental health/behavioural	Substance abuse	E24.4, F10-F19, F55*, G24.0*, G31.2, G40.5, G62.1, G72.0, G72.1, I42.6, K29.2, K70, K85.2, K85.3, K86.0, O35.4, R78.1*-R78.5*, Y47, Y49, Z50.2, Z50.3, Z71.4, Z71.5, Z72.2*, Z86.4
	Self-harm	X60-X84, Y10-Y34†, Y87.0, Y87.2†, Z91.5
	Other mental health problems	F00-F01, F02.8, F03-F09, F20-F48, F50, F53, F54, F59*, F60-F69, F99*, Z09.3*, Z50.4*, Z86.5, Z91.4*
	Behavioural/developmental disorders	F70-F79, F80.0-F80.2, F80.8, F80.9, F81-F84, F88, F89, F90-F98
Cancer/blood disorders	Neoplasms	C00-C97, D00-D02, D05-D09, D12, D13, D14.1-D14.4, D15, D20, D32-D35, D37-D48, D63.0, E34.0, E88.3, G13.0, G13.1, G53.3, G55.0, G63.1, G73.1, G73.2, G94.1, M36.0, M36.1, M49.5, M82.0, M90.6, M90.7, N08.1, N16.1, Y43.1-Y43.3, Y84.2, Z08, Z51.0-Z51.2, Z54.1, Z54.2, Z85, Z86.0, Z92.3
	Immunological disorders	D80-D84, G53.2, Q98.0
	Anaemia and other blood disorders	D50*, D56.0-D56.2, D56.4, D56.8, D56.9, D57.0-D57.2, D57.8, D58, D61.0, D61.9, D64*, D66, D67, D68.0-D68.2, D68.4-D68.9, D69, D70-D76, M36.2-M36.4, M90.4, N08.2, Z86.2
Chronic infections	HIV	B20-B24, F02.4, R75, Z21
	Tuberculosis	A15-A19, E35.0, K23.0, K67.3, K93.0, M01.1, M49.0, P37.0
	Other	A50, A81, B18, B37.1, B37.5, B37.6, B37.7, B38.1, B39.1, B40.1, B44.0, B44.7, B45, B46, B48.7, B50.0, B50.8*, B51.0, B51.8*, B52.8*, B52.0, B55, B57.2-B57.5, B58.0, B59, B67, B69, B73, B74, B78.7, B90-B94, F02.1, K23.1, K93.1, M00, N33.0, P35.0-P35.2, P35.8, P35.9, P37.1
Respiratory	Asthma and chronic lower respiratory disease	J41-J47
	Cystic fibrosis	E84, P75
	Injuries	S17*, S27*, S28*, T27*, T91.4*
	Congenital anomalies	Q30-Q37, Q79.0
	Other	G47.3, J60-J70, J80-J86, J96.1, J98, P27, Y55.6, Z43.0, Z93.0, Z94.2

Metabolic/endocrine/digestive/renal/ genitourinary	Diabetes	E10-E14, G59.0, G63.2, I79.2, M14.2, N08.3, O24, Y42.3
	Other endocrine	E00, E03.0, E03.1, E07.1, E22.0, E23.0, E25, E26.8, E29.1, E31, E34.1, E34.2, E34.5, E34.8, G13.2, G73.5, Y42.1
	Metabolic	D55, E70-E72, E74-E78, E79.1-E79.9,E80.0-E80.3, E80.5, E80.7, E83, E85, E88.0, E88.1, E88.2*, E88.8, E88.9, G73.6, L99.0, M14.4, M14.3, N16.3
	Digestive	K20, K21.0, K22, K23.8, K25-K28, K29.0, K29.1, K29.3-K29.9, K31, K50-K52, K55, K57, K59.2, K63.0-K63.3, K66, K72-K76, K80-K83, K85.0, K85.1, K85.8, K85.9, K86.1-K86.9, K87.0, K90, M07.4, M07.5, M09.1, M09.2, T86.4, Z43.2-Z43.4, Z46.5, Z90.3, Z90.4, Z93.2-Z93.5
	Renal/GU	D63.8, G63.8, G99.8, I68.8, M90.8, N08.4, N00-N05, N07, N11-N15, N16.0, N16.2, N16.4, N16.5, N16.8, N18, N19, N20-N23, N25, N26, N28, N29, N31, N32, N33.8, N35, N36, N39.1, N39.3, N39.4, N40-N42, N70-N74, N80-N82, N85, N86*, N87,N88, P96.0, T82.4, T83.1, T83.2, T83.4-T83.9, T85.5, T86.1, Y60.2, Y61.2, Y62.2, Y84.1, Z49, Z93.6, Z94.0, Z99.2
	Congenital anomalies of the digestive/renal/GU system	Q38.0, Q38.3, Q38.4, Q38.6-Q38.8, Q39, Q40.2, Q40.3, Q40.8, Q40.9, Q41, Q42, Q43.1, Q43.3-Q43.7, Q43.9, Q44, Q45, Q50.0, Q51, Q52.0-Q52.2, Q52.4, Q54.0-Q54.3, Q54.8, Q54.9, Q55.0, Q55.5, Q56, Q60.1, Q60.2, Q60.4-Q60.6, Q61, Q62.0-Q62.6, Q62.8, Q63.0-Q63.2, Q63.8, Q63.9, Q64, Q79.2-Q79.5, Q87.8, Q89.1, Q89.2
	Injuries	S36*, S37*, S38*, S39.6*, S39.7*, T06.5*, T28*, T91.5*
	Other/unspecific	E66, G63.3, G99.0, M14.5, N92*, Z86.3, Z93.8
Musculoskeletal/skin	Musculoskeletal/connective tissue	G55.1-G55.3, G63.5, G63.6, G73.7, J99.0, J99.1, L62.0, M05, M06, M07.0-M07.3, M07.6, M08, M09.8, M10-M13, M14.0, M14.6, M14.8, M30-M35, M40-M43, M45-M48,M50-M54, M60-M62, M63.8, M80.1-M80.9, M81.1-M81.9, M82.1, M82.8, M84.0-M84.2, M84.8, M84.9, M85, M86.3-M86.6, M89, M90.0, M91-M94, N08.5, Y45.4
	Skeletal injuries/amputations	S13*, S22.0*-S22.2*, S22.5*, S23*, S32*, S33*, S68.3*, S68.4*, S68.8*, S77*, S78*, S87*, S88*, S97*, S98.0*, S98.2*-S98.4*, T02*, T04*, T05*, T20.3*, T20.7*, T21.3*, T21.7*, T22.3*, T22.7*, T23.2*, T23.3*, T23.6*, T23.7*, T24.3*, T24.7*, T25.2*, T25.3*, T25.6*, T25.7*, T29.3*, T29.7*, T30.3*, T30.7*, T31.2*-T31.9*, T32.2*-T32.9*, T87.3-T87.6, T91.2* T91.8*, T92.6*, T93.1*, T93.4*, T93.6*, T94.0*, T94.1*, T95.0*, T95.1*, T95.4*, T95.8*, T95.9*, Y83.5, Z89.1, Z89.2, Z89.5-Z89.8, Z97.1
	Chronic skin disorders	L10, L11.0, L11.8, L11.9, L12-L14, L28, L40-L45, L57, L58.1, L59, L87, L88, L90, L92, L95, L93, L98.5, M09.0, Q80, Q81, Q87.0-Q87.5, Q89.4

	Congenital anomalies	Q18.8, Q65.0-Q65.2, Q65.8, Q65.9, Q67.5, Q68.2, Q68.3*-Q68.5*, Q71-Q73, Q74, Q75.3-Q75.9, Q76.1-Q76.4, Q77, Q78, Q79.6, Q79.8, Q82.0-Q82.4, Q82.9, Q86.2, Q89.7-Q89.9
Neurological	Epilepsy	F80.3, G40.0-G40.4, G40.6-G40.9, G41, R56.8, Y46.0-Y46.6
	Cerebral palsy	G80-G83
	Injuries of brain, nerves, eyes or ears	S05*-S08*, S12*, S14*, S24*, S34*, S44*, S54*, S64*, S74*, S84*, S94*, T06.0*-T06.2*, T26*, T90.4*, T90.5*, T91.1*, T91.3*, T92.4*,
	Chronic eye conditions	H05.1-H05.9, H13.3, H17, H18, H19.3, H19.8, H21, H26, H27, H28.0-H28.2, H31, H32.8, H33, H34, H35, H40, H42.0, H43, H44, H47, H54.0- H54.2, H54.4, T85.2, T85.3, Z44.2
	Chronic ear conditions	H60.2, H65.2-H65.4, H66.1-H66.3, H69.0, H70.1, H73.1, H74.0-H74.3, H75.0, H80, H81.0, H81.4, H83.0, H83.2, H90.0, H90.3, H90.5, H90.6, H91, Z45.3
	Perinatal conditions	P10, P21.0, P52, P57, P90, P91.1, P91.2, P91.6
	Congenital anomalies of neurological or sensory systems	Q00-Q07, Q10.4, Q10.7, Q11-Q12, Q13.0-Q13.4, Q13.8, Q13.9, Q14-Q16, Q75.0, Q75.1, Q85, Q86.0, Q86.1, Q86.8, Q90-Q93, Q95.2, Q95.3, Q97, Q99
	Other	F02.2, F02.3,G00-G09, G10-G12, G13.8, G14, G20-G23, G24.1-G24.9, G25-G30, G31.0-G31.1, G31.8, G31.9, G32-G37, G43-G46, G47.0-G47.2, G47.4-G47.9, G50-G52, G53.0, G53.1, G53.8, G54, G55.8, G56-G58, G59.8, G60, G61, G62.0, G62.2-G62.9, G64, G70, G71,G72.2-G72.9, G73.0, G73.3, G90-G93, G94.2, G94.8, G95, G96, G98, G99.1, G99.2, I60-I67, I68.0, I68.2, I69, I72.0, I72.5, T85.0, T85.1, Y46.7-Y46.8, Z98.2
Cardiovascular	Congenital heart disease	Q20-Q26, Q89.3
	Other	I00*-I28*, I31*-I39*, I41*, I42.0*-I42.5*, I42.7*-I42.9*, I43.0*, I43.1, I43.2*-I43.8*, I44.1*-I44.7*, I45.1*-I45.9*, I46*-I51*, I52.8, I70*-I71*, I72.1*-I72.4*, I72.8*, I72.9*, I73*-I77*, I79.0*, I79.1*, I79.8*, I81*-I82*, I98*-I99*, M03.6, N08.8, Q27, Q28, S26*, T82.0-T82.3, T82.5-T82.9, T86.2, Y60.5, Y61.5, Y62.5, Y84.0, Z45.0, Z50.0, Z94.1, Z95
Codes indicating non-specific chronic condition	-	R62, R63.3, Z43.1, Z51.5, Z75.5, Z93.1, Z99.3

*Codes used with severity criteria: length of stay of hospital admission mentioning code >3 days and the discharge date of the admission where the code is more than 30 days before death.

†Codes used with age criteria: Age at death (if from death certificates) or age at admission (if from hospital records) must be 10 years or older.
